# Ecosystem carbon use efficiency in ecologically vulnerable areas in China: Variation and influencing factors

**DOI:** 10.3389/fpls.2022.1062055

**Published:** 2022-12-12

**Authors:** Zhaogang Liu, Zhi Chen, Guirui Yu, Meng Yang, Weikang Zhang, Tianyou Zhang, Lang Han

**Affiliations:** ^1^ Key Laboratory of Ecosystem Network Observation and Modeling, Institute of Geographic Sciences and Natural Resources Research, Chinese Academy of Sciences, Beijing, China; ^2^ College of Resources and Environment, University of Chinese Academy of Sciences, Beijing, China; ^3^ Yanshan Earth Critical Zone and Surface Fluxes Research Station, University of Chinese Academy of Sciences, Beijing, China; ^4^ College of Grassland Agriculture, Northwest A&F University, Yangling, China; ^5^ Institute of Surface-Earth System Science, School of Earth System Science, Tianjin University, Tianjin, China

**Keywords:** ecologically vulnerable areas, carbon use efficiency, grassland, eddy covariance, climate change

## Abstract

Ecologically vulnerable areas (EVAs) are regions with ecosystems that are fragile and vulnerable to degradation under external disturbances, e.g., environmental changes and human activities. A comprehensive understanding of the climate change characteristics of EVAs in China is of great guiding significance for ecological protection and economic development. The ecosystem carbon use efficiency (CUEe) can be defined as the ratio of the net ecosystem productivity (NEP) to gross primary productivity (GPP), one of the most important ecological indicators of ecosystems, representing the capacity for carbon transfer from the atmosphere to a potential ecosystem carbon sink. Understanding the variation in the CUEe and its controlling factors is paramount for regional carbon budget evaluation. Although many CUEe studies have been performed, the spatial variation characteristics and influencing factors of the CUEe are still unclear, especially in EVAs in China. In this study, we synthesized 55 field measurements (3 forestland sites, 37 grassland sites, 6 cropland sites, 9 wetland sites) of the CUEe to examine its variation and influencing factors in EVAs in China. The results showed that the CUEe in EVAs in China ranged from -0.39 to 0.67 with a mean value of 0.20. There were no significant differences in the CUEe among different vegetation types, but there were significant differences in CUEe among the different EVAs (agro-pastoral ecotones < Tibetan Plateau < arid and semiarid areas < Loess Plateau). The CUEe first decreased and then increased with increasing mean annual temperature (MAT), soil pH and soil organic carbon (SOC) and decreased with increasing mean annual precipitation (MAP). The most important factors affecting the CUEe were biotic factors (NEP, GPP, and leaf area index (LAI)). Biotic factors directly affected the CUEe, while climate (MAT and MAP) and soil factors (soil pH and SOC) exerted indirect effects. The results illustrated the comprehensive effect of environmental factors and ecosystem attributes on CUEe variation, which is of great value for the evaluation of regional ecosystem functions.

## Introduction

Ecologically vulnerable areas (EVAs), also denoted as ecological ecotones, refer to the transitional areas at the intersection of two or more ecosystems, and are mainly located in the ecotones of different ecosystems, such as areas exhibiting agriculture, animal husbandry, forestland, and grassland (Yu et al., 2017; Feng et al., 2022; Hu et al., 2022). Environmental and biotic factors in EVAs occur in a critical state of phase transition. These ecotones are characterized by a low anti-interference ability, sensitivity to climate change, notable temporal and spatial fluctuations, significant marginal effect, and high environmental heterogeneity. China is one of the countries with the largest distribution area of EVAs, the largest number of vulnerable ecological types, and the most obvious ecological vulnerability worldwide (Yu et al., 2017). EVAs above the moderate level account for 55% of the total land area of China (Hu et al., 2022). We mainly focused on the following four types of EVAs: agro-pastoral ecotones, Tibetan Plateau, arid and semiarid areas, and Loess Plateau. Comprehensively understanding the characteristics of climate change in EVAs in China is of great significance for ecological protection and economic development.

The ecosystem carbon use efficiency (CUEe) can be defined as the ratio of the net ecosystem productivity (NEP) to the gross primary productivity (GPP) (Fernández-Martínez et al., 2014a; Manzoni et al., 2018). This index can be used to describe the level of total carbon stored and obtained by a given ecosystem from the atmosphere, and represents the potential carbon sink capacity of the ecosystem (Fernández-Martínez et al., 2014a). This quantity plays a very important role in the ecosystem productivity model (Fernández-Martínez et al., 2014a; Sinsabaugh et al., 2017). In addition, the efficiency of ecosystems in transforming the GPP into plant and soil storage largely determines the carbon sequestration capacity of terrestrial ecosystems and its feedback to climate change (Baldocchi, 2014). Therefore, identifying the characteristics of the CUEe and its influencing factors in EVAs could facilitate a greater understanding of the trend of global carbon cycle change within the context of climate change and provide a basis for vegetation carbon sink management.

At present, many studies use remote sensing to study the CUEe, but different studies provide very different estimates of the CUEe (Curtis et al., 2005; Manzoni et al., 2018; Chen et al., 2019). Therefore, it is necessary to use direct observation data to analyze the CUEe and its influencing factors to provide support for future model revision and accurate CUEe simulation (Liu et al., 2020). In addition, most studies focused on the vegetation carbon use efficiency (CUE) and microorganism CUE (Chen et al., 2019), but there is less CUEe research. By integrating published literature on carbon flux observations based on the eddy covariance method, An et al. (2017) found that the CUEe in grassland and forestland was consistent, while other studies found that the CUEe in grassland was higher than that in forestland (Law et al., 2002). The CUEe is also affected by environmental conditions (Luyssaert et al., 2007; Bradford and Crowther, 2013; Fernández-Martıńez et al., 2014b). It has been found that the main factor affecting the grassland CUEe is the mean annual precipitation (MAP), which is linearly negatively correlated with the CUEe (Hirata et al., 2008; Zhang et al., 2009). Chen et al. (2019) found that the temperature was the main controlling factor of the forestland CUEe. Although many CUEe studies have been performed by predecessors, the spatial variation characteristics and influencing factors of the CUEe are still unclear, especially in EVAs in China.

We used eddy-covariance carbon fluxes measurements of 55 ecosystems in EVAs in China. The following topics are expected to be addressed: (1) determine of the spatial variation pattern of the CUEe, and (2) analysis of the influencing factors of the CUEe and its regulatory mechanism.These findings could help us to better understand the regional carbon balance under climate change and strengthen the management and restoration of EVAs in China.

## Materials and methods

### Gross Primary Productivity (GPP) and Net Primary Productivity (NEP) data collection and screening

We collected gross primary productivity (GPP) and net primary productivity (NEP) data measured *via* the eddy covariance method from literature published over the past 20 years (2002-2019) in regard to EVAs in China. Based on Web of Science database (http://apps.webofknowledge.com) and CNKI database (http://www.cnki.net), data were retrieved by using “eddy covariance”, “carbon flux” “carbon exchange”, “carbon budget”, “productivity”, “gross primary productivity”, “net ecosystem productivity” and “net ecosystem exchange (NEE)” as keywords. The data were filtered and corrected by researchers at each site, using coordinate rotation, WPL correction, storage flux calculation, outlier filtering, nighttime flux correction, NEE gap filling and partitioning. Additionally, the data were continuously measured for at least an entire year. At the same time, the geographic location, ecosystem and vegetation type at each observation site were extracted. The CUEe value was estimated as CUEe=NEP/GPP.

**Figure 1:**
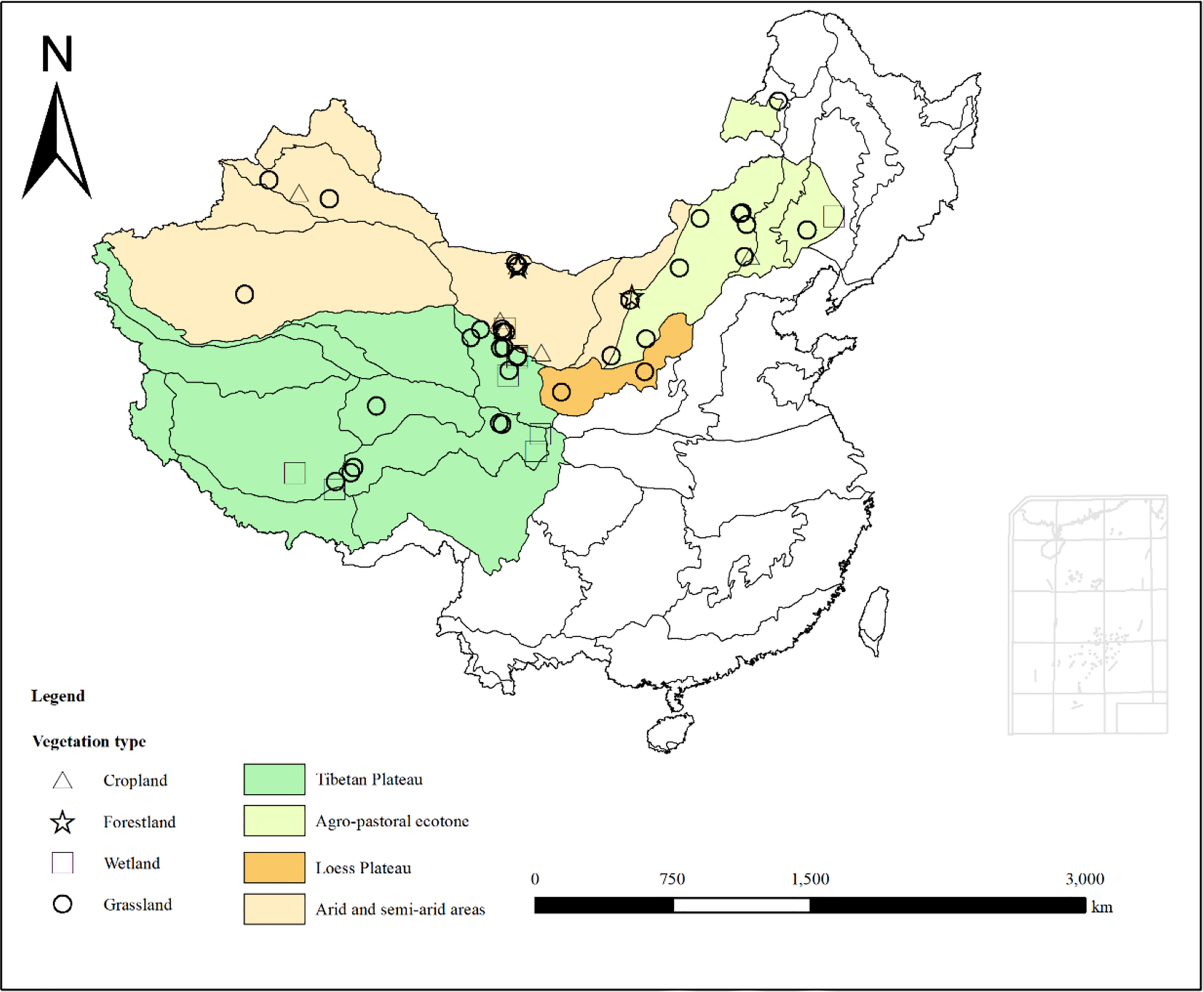
Distribution of flux sites in ecologically vulnerable areas (EVAs) in China.

Through the above standard screening approach, carbon fluxes observation data of 55 ecosystems covering forestlands, grasslands, croplands and wetlands were obtained (**Figure 1**). The data covered the temperate zone, warm temperate zone, Tibetan Plateau and other climatic regions. The latitude range of the selected flux stations was 30.47°N-49.35°N, and the longitude range was 83.57°E-122.65°E. There were 37 grassland sites, 3 forestland sites, 9 wetland sites and 6 cropland sites (**Table 1**).

**Table 1:** Site information in this study.

Site	Latitude (°N)	Longitude (°E)	Vegetation type	Ecotone	MAT^†^ (°C)	MAP^†^ (mm)	Observation year
Dangxiong wetland	30.47	91.07	Wetland	Tibetan Plateau	2.96	420	2009-2013
Dangxiong grassland	30.85	91.08	Grassland	Tibetan Plateau	-1.98	416	2004-2011
Shenzha	30.95	88.68	Wetland	Tibetan Plateau	1.89	385	2016-2019
Naqu grassland1	31.37	91.90	Grassland	Tibetan Plateau	-0.38	426	2008-2008
Naqu grassland2	31.64	92.01	Grassland	Tibetan Plateau	-1.60	430	2012-2017
Ruoergai1	33.10	102.65	Wetland	Tibetan Plateau	2.37	694	2013-2017
Ruoergai2	33.93	102.87	Wetland	Tibetan Plateau	1.90	654	2008-2009
Sanjiangyuan degraded	34.35	100.55	Grassland	Tibetan Plateau	-3.24	590	2006-2008
Sanjiangyuan	34.41	100.40	Grassland	Tibetan Plateau	-1.61	552	2005-2008
Fenghuoshan	34.72	92.89	Grassland	Tibetan Plateau	-6.01	301	2015-2015
SACOL	35.95	104.13	Grassland	Loess Plateau	7.88	348	2007-2012
Qinghai wetland	36.70	100.78	Wetland	Tibetan Plateau	0.85	418	2011-2015
Ansai	36.86	109.32	Grassland	Loess Plateau	9.68	490	2012-2014
Haiyan	36.95	100.85	Grassland	Tibetan Plateau	-0.08	435	2010-2010
Qinghai lake	37.58	101.33	Wetland	Tibetan Plateau	-1.78	465	2007-2016
Haibei grassland	37.62	101.32	Grassland	Tibetan Plateau	-2.07	469	2002-2004
Haibei shrubland	37.67	101.33	Grassland	Tibetan Plateau	-2.61	475	2003-2012
Haibei wetland	37.68	101.31	Wetland	Tibetan Plateau	-2.84	475	2003-2006
Yanchi	37.71	107.23	Grassland	Arid and semiarid areas	7.97	309	2012-2016
Hexi	37.87	102.83	Cropland	Arid and semiarid areas	7.99	167	2014-2018
Yakou	38.01	100.24	Grassland	Tibetan Plateau	-7.73	457	2015-2016
Arou	38.05	100.45	Grassland	Tibetan Plateau	-1.98	404	2009-2016
Shule	38.42	98.32	Grassland	Tibetan Plateau	-6.69	300	2008-2012
Yulin	38.45	109.47	Grassland	Agro-pastoral ecotone	7.92	376	2011-2012
Huazaizi	38.77	100.32	Grassland	Arid and semiarid areas	6.84	264	2012-2012
Shenshawo	38.79	100.49	Grassland	Arid and semiarid areas	7.38	215	2012-2012
Dashalong	38.84	98.94	Grassland	Tibetan Plateau	-6.89	342	2013-2016
Daman	38.86	100.37	Cropland	Arid and semiarid areas	6.91	220	2012-2018
Bajitan	38.92	100.30	Grassland	Arid and semiarid areas	7.41	211	2014-2014
Tazhong.	38.96	83.65	Grassland	Arid and semiarid areas	11.96	31	2009-2013
Zhangye	38.98	100.45	Wetland	Arid and semiarid areas	7.67	188	2012-2014
Linze	39.32	100.13	Cropland	Arid and semiarid areas	7.89	161	2008-2008
Kubuqi grassland	40.38	108.55	Grassland	Arid and semiarid areas	7.03	228	2006-2006
Kubuqi forestland	40.54	108.69	Forestland	Agro-pastoral ecotone	7.44	227	2005-2006
Siziwang fenced	41.79	111.89	Grassland	Agro-pastoral ecotone	3.53	216	2010-2011
Siziwang grazing	41.79	111.90	Grassland	Agro-pastoral ecotone	3.50	219	2010-2011
Qidaoqiao	41.98	101.17	Forestland	Arid and semiarid areas	8.36	35	2013-2016
Heihe mixed forestland	41.99	101.13	Forestland	Arid and semiarid areas	8.21	40	2013-2013
Heihe-luodi	42.00	101.13	Grassland	Arid and semiarid areas	8.18	41	2012-2012
Sidaoqiao	42.00	101.14	Grassland	Arid and semiarid areas	8.26	40	2013-2014
Heihe cropland	42.00	101.13	Cropland	Arid and semiarid areas	8.26	40	2013-2013
Duolun cropland	42.05	116.67	Cropland	Agro-pastoral ecotone	3.23	409	2005-2006
Duolun grassland	42.05	116.28	Grassland	Agro-pastoral ecotone	3.05	400	2005-2006
Heihe desert	42.11	100.99	Grassland	Arid and semiarid areas	8.73	34	2015-2015
Naiman	42.92	120.70	Grassland	Agro-pastoral ecotone	7.15	432	2015-2017
Keerqin	43.34	122.65	Wetland	Agro-pastoral ecotone	7.02	474	2016-2016
Xinlinhot fenced	43.55	116.67	Grassland	Agro-pastoral ecotone	1.03	320	2006-2008
Xinlinhot degraded	43.55	116.67	Grassland	Agro-pastoral ecotone	1.03	320	2006-2006
Xilinguole	44.08	113.57	Grassland	Agro-pastoral ecotone	2.62	198	2008-2010
Xinlinhot stipa	44.13	116.33	Grassland	Agro-pastoral ecotone	1.84	274	2004-2006
Maodeng	44.16	116.49	Grassland	Agro-pastoral ecotone	1.46	284	2013-2017
Wulanwusu	44.28	85.82	Cropland	Arid and semiarid areas	7.34	140	2009-2013
Fukang	44.28	87.93	Grassland	Arid and semiarid areas	6.69	174	2002-2012
Aibi lake	44.62	83.57	Grassland	Arid and semiarid areas	9.02	158	2012-2015
Hulunbeier	49.35	120.10	Grassland	Agro-pastoral ecotone	-2.51	369	2009-2010

^†^MAP, mean annual precipitation; MAT, mean annual temperature.

### Climate, vegetation and soil data collection

Climatic variables including the mean annual temperature (MAT) and mean annual precipitation (MAP) were also collected. The data were derived from the same studies as the carbon fluxes data. Mean values of the air temperature and precipitation in the observation year were calculated as the MAT and MAP, respectively.

The leaf area index (LAI) was derived from the satellite-borne Moderate Resolution Imaging Spectroradiometer (MODIS) data product (MOD13Q1) with a spatial resolution of 1 km and a temporal resolution of 8 days from 2000 to 2018. Soil data including the soil pH and soil organic carbon content (SOC) were retrieved from the global normalized soil dataset of the Harmonized World Soil Database (version 1.2) (https://daac.ornl.gov/cgi-bin/dsviewer.pl?ds_id=1247).

### Statistical analyses

First, we compared differences in the CUEe among the different EVAs and vegetation types in China. The relationship between the GPP and NEP, and the relationships between the CUEe and longitude and latitude were analyzed *via* linear regression.

Linear and quadratic regression analyses were performed to examine the correlation between the MAT, MAP, soil pH and SOC and the CUEe with a significance level of α = 0.05. The hierarchical partitioning method was employed to determine the contributions of the longitude, latitude, MAT, MAP, soil pH, SOC, GPP and NEP to the CUEe *via* the “rdacca.hp” package in R (Lai et al., 2022).

We further established a structural equation model (SEM) to evaluate the direct and indirect factors regulating the CUEe, and assessed their contributions to the total effects of standardization (direct effects plus indirect effects). The causal relationship between the predicted variables was based on *a priori* knowledge of the effects of climatic variables (MAT and MAP), geographic location (longitude and latitude), soil parameters (soil pH and SOC), LAI, GPP and NEP on the CUEe. Since the variables of climate, geographic location and soil groups were closely related, principal component analysis (PCA) was conducted to create a multivariate index representing each group (Wang et al., 2017). The first principal component (PC1) explained 61-80% of the total variance of each group and was subsequently used for SEM analysis, in which the data were fitted to the model using the maximum likelihood estimation method. The model’s adequacy was determined using the χ^2^ test method, goodness of fit (GFI) index, and root mean squared error of approximation (RMSEA) index. Favorable model fits were indicated by no significant difference when using the χ^2^-test method (*P* > 0.05), a high GFI value (>0.9), and a low RMSEA value (<0.08) (Liu et al., 2017). SEM analysis was conducted in Amos 21.0 (Amos Development Corporation, Chicago, IL).

All analyses were conducted in R software (version 3.5.1, R Development Core Team, Vienna, Austria). ArcGis 10.1 and R were used for plotting.

## Results

### Variation characteristics and spatial pattern of the Ecosystem Carbon Use Efficiency (CUEe)

**Figure 2:**
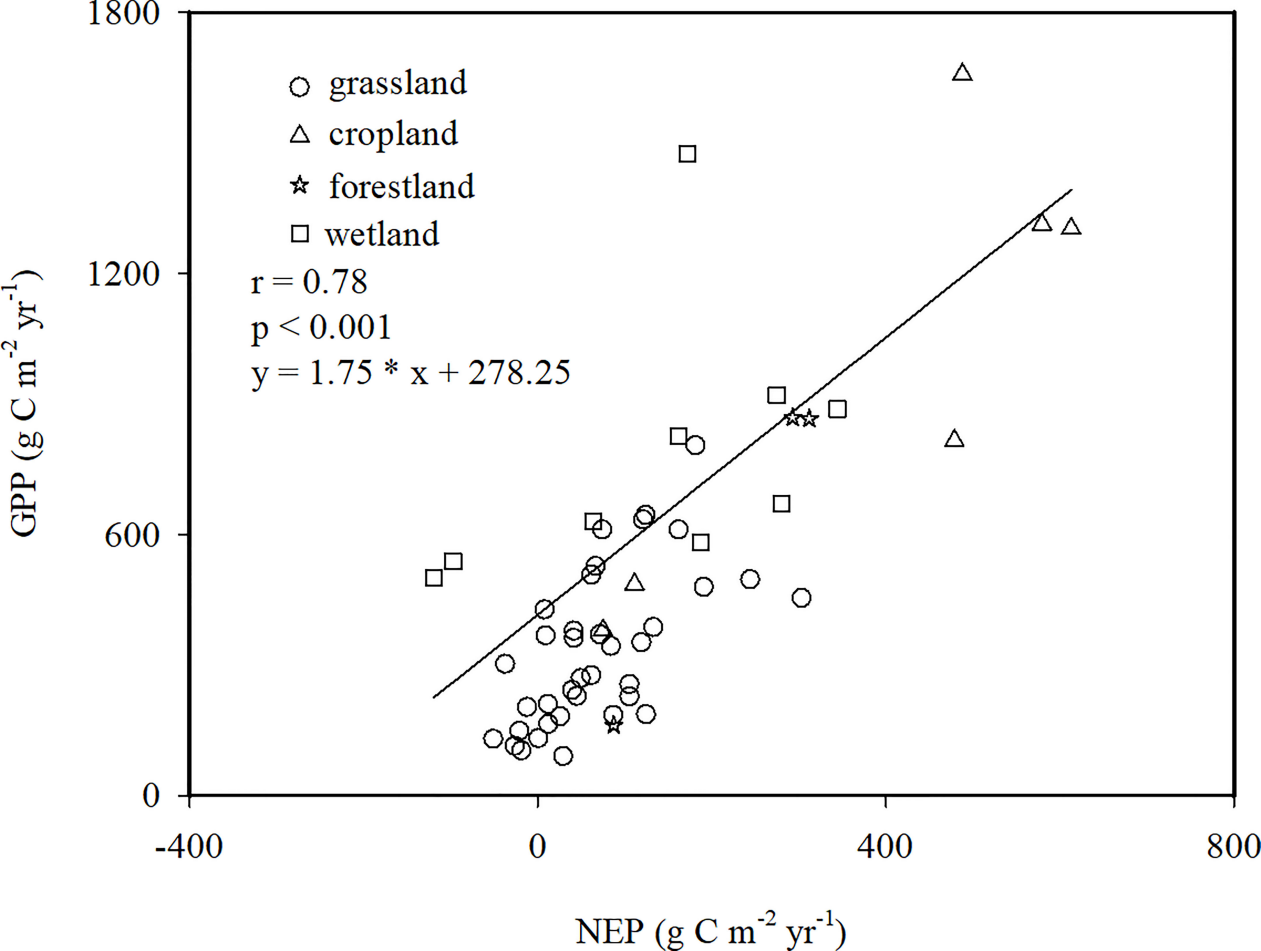
Relationship between the net ecosystem productivity (NEP) and gross primary productivity (GPP) in ecologically vulnerable areas (EVAs) in China.

The results showed that the GPP ranged from 91.25 g C m^-2^ yr^-1^ in the Bajitan grassland to 1657.9 g C m^-2^ yr^-1^ in the Linze cropland (**Figure 2**). The NEP and GPP of the different ecosystems were linearly correlated (**Figure 2**). The CUEe varied greatly among the different ecosystems, such as -0.39 in the Xilinhot Stipa grassland and 0.67 in the Ansai grassland. Based on the site average, the estimated average value of the CUEe of EVAs in China was 0.20 (**Figure 2**).

**Figure 3:**
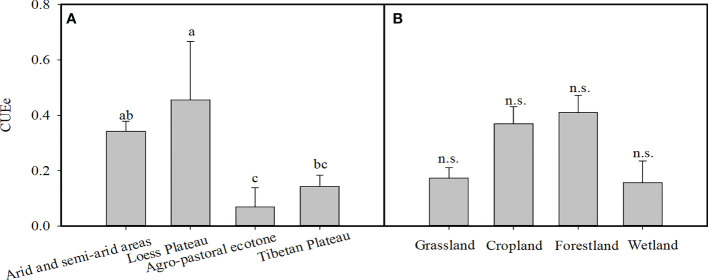
**(A)** Variation in the ecosystem carbon use efficiency (CUEe) in different ecologically vulnerable areas (EVAs) and **(B)** vegetation types in China. The different lowercase letters indicate significant differences at the p < 0.05 level for the CUEe among the different EVAs in China, n.s. indicates no significant differences at the p < 0.05 level for the CUEe among the different vegetation types.

**Figure 4:**
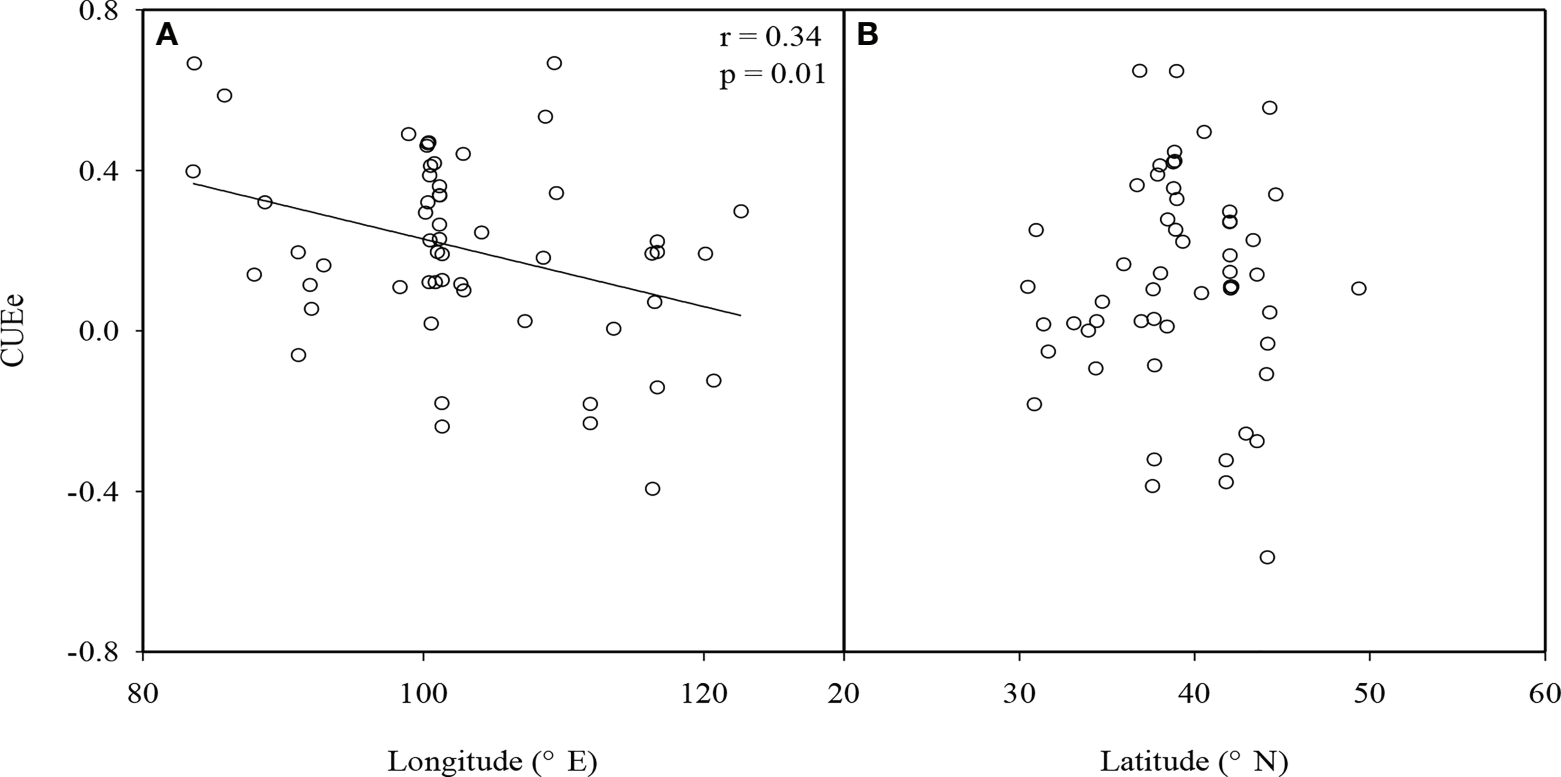
**(A)** Relationship between the ecosystem carbon use efficiency (CUEe) and longitude and **(B)** latitude in ecologically vulnerable areas (EVAs) in China.

We found that there were significant differences in the CUEe among the different EVAs in China (p<0.05). The average values of the CUEe in arid and semiarid areas, Loess Plateau, agro-pastoral ecotones, and Tibetan Plateau were 0.34, 0.46, 0.07, and 0.14, respectively. Among them, the CUEe on the Loess Plateau was the highest, and that in the agro-pastoral ecotones was the lowest (**Figure 3**). There was no significant difference in the CUEe among the different vegetation types (p>0.05), in which the CUEe values in grassland, cropland, forestland and wetland areas were 0.17, 0.37, 0.41 and 0.16, respectively (**Figure 3**). The CUEe significantly decreased with increasing longitude, while it showed no trend with increasing latitude (**Figure 4**).

### Impact of climate and soil factors on the Ecosystem Carbon Use Efficiency (CUEe)

**Figure 5:**
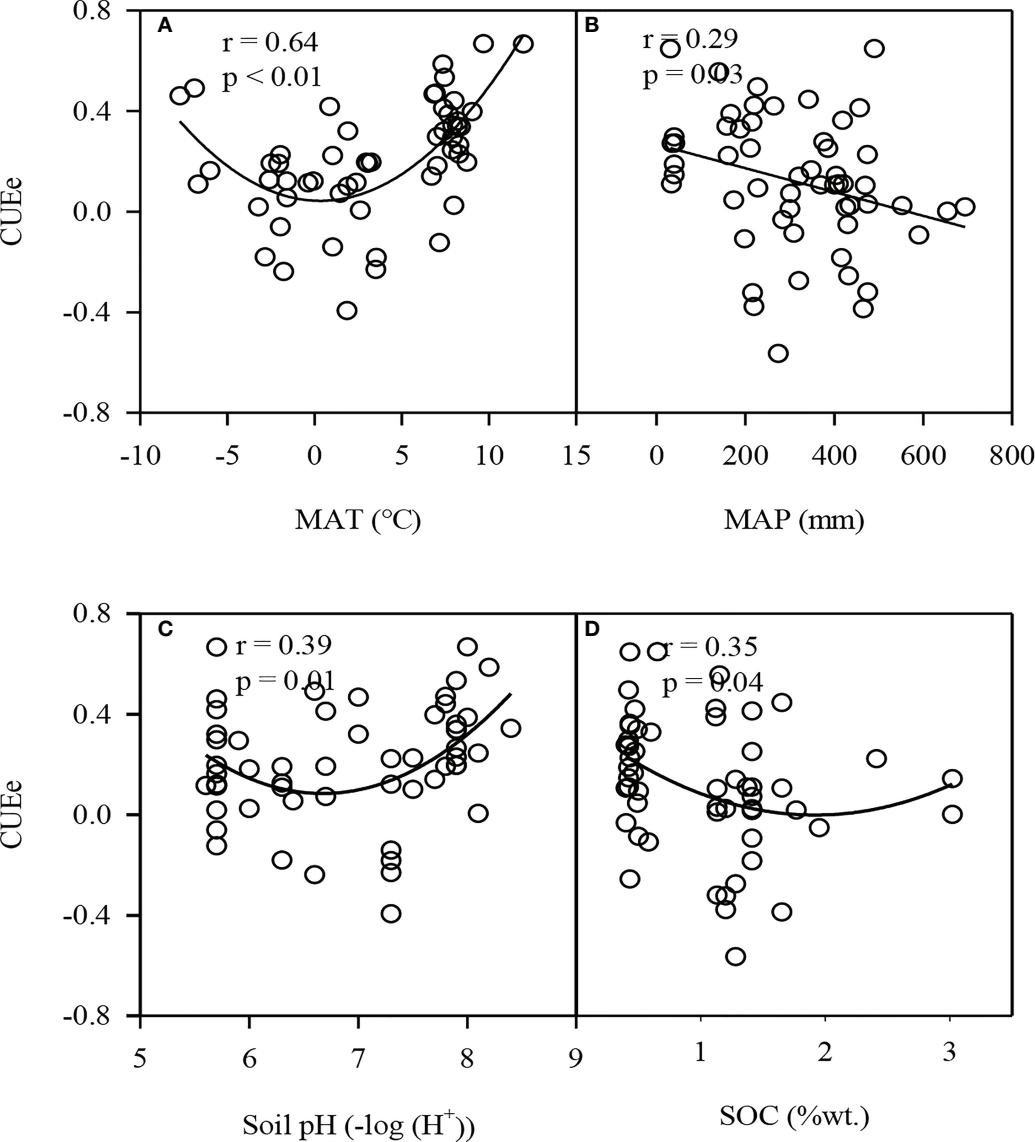
**(A)** Relationship between the ecosystem carbon use efficiency (CUEe) and MAT, **(B)** MAP, **(C)** Soil pH and **(D)** SOC in ecologically vulnerable areas (EVAs) in China. MAT, mean annual temperature; MAP, mean annual precipitation; SOC, soil organic carbon.

We mainly analyzed the impact of climate factors (MAT and MAP) and soil factors (soil pH and SOC) on the CUEe. The CUEe first decreased and then increased with increasing MAT, soil pH and SOC, and decreased with increasing MAP (**Figure 5**). Among the four environmental factors, MAT exerted the largest impact on the CUEe, which could explain nearly 41% of the variation in the CUEe.

### Regulation mechanism of the Ecosystem Carbon Use Efficiency (CUEe)

**Figure 6:**
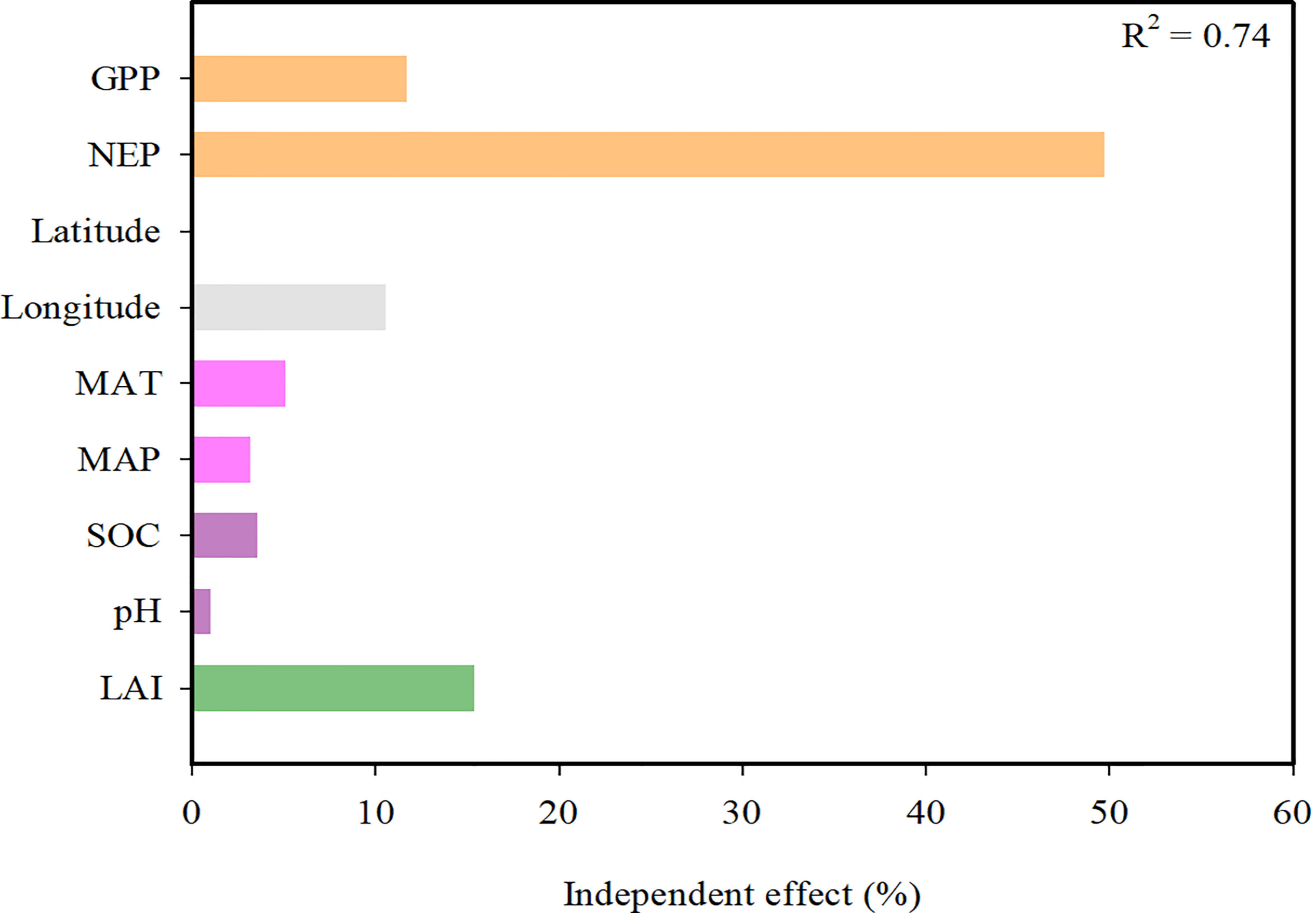
Hierarchical partitioning analysis between the explanatory variables and ecosystem carbon use efficiency (CUEe). MAT, mean annual temperature; MAP, mean annual precipitation; LAI, leaf area index; SOC, soil organic carbon; NEP, net ecosystem productivity; GPP, gross primary productivity.

Hierarchical partitioning analysis showed that the NEP, LAI and GPP were the most important factors influencing the CUEe, followed by the longitude (**Figure 6**). It is not difficult to determine that compared to the soil factors (SOC and soil pH), the climate factors (MAT and MAP) exerted a greater impact on the CUEe.

**Figure 7:**
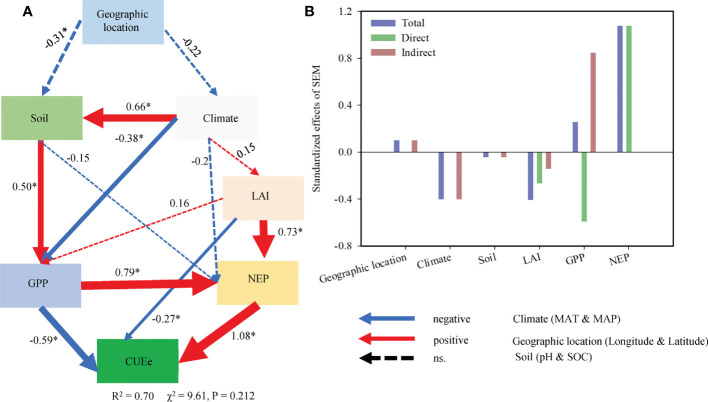
**(A)** Structure equation modeling exploring the direct and indirect effects of the different factors on the ecosystem carbon use efficiency (CUEe) and **(B)** standardized effects of the different factors on the CUEe. The blue and red arrows indicate negative and positive relationships, respectively. The dashed line represents a nonsignificant relationship (p > 0.05). The arrow width is proportional to the strength of the relationship. The numbers adjacent to the arrows are standardized path coefficients. * indicates the significance level is less than 0.05. MAT, mean annual temperature; MAP, mean annual precipitation; LAI, leaf area index; SOC, soil organic carbon; NEP, net ecosystem productivity; GPP, gross primary productivity.

SEM analysis showed that the GPP, NEP and LAI directly affected the CUEe, while climate and soil factors exerted indirect effects. Jointly considering the direct and indirect effects, biotic factors (GPP, NEP and LAI) were the most important predictors determining the regional variation in the CUEe (**Figure 7**). Whether through hierarchical partitioning analysis or SEM, the results showed that these variables could explain approximately 70% of the total variation in the CUEe. Regarding the CUEe, the NEP exerted a greater impact on the CUEe than the GPP (**Figures 6**, **7**).

## Discussion

### Spatial variation in the CUEe in ecologically vulnerable areas in China

Many studies have found that the CUE of plants is a constant (Waring et al., 1998; Delucia et al., 2007). We provided a reference for the basic status of the CUEe in EVAs in China, and suggested that the CUEe value ranged from -0.39 to 0.67 (**Figure 2**). The variation range of the CUEe in this study was larger than that in other studies (Chen et al., 2018); the variation range of the CUEe values in EVAs remained reasonable and was smaller than the variation range from −1 to 0.6 for global ecosystems (Chen et al., 2015b). The increased variability of the CUEe may be due to the significant deviation in heterotrophic respiration (Rh) and its ratio to the net primary productivity (NPP) (Chen et al., 2018). The lowest CUEe value was found in the Xilinhot Stipa grassland, where the large amount of autotrophic respiration (Ra) and Rh release exceeded the low GPP. The highest CUEe value was found in the Ansai grassland, indicating that the carbon consumption of ecosystem respiration was low on the Loess Plateau. According to the site average value, the average CUEe value of EVAs in China was estimated at 0.2, which indicated that an average productivity of 20% was fixed in ecosystem biomass and soil organic matter (Hursh et al., 2017). This efficiency was higher than the average CUEe value in other Asian countries and global ecosystems (Kato and Tang, 2008; Chen et al., 2015b).

The CUEe varied with the different ecosystem vegetation composition and structure. Gilmanov et al. (2010) found that the CUEe in European grasslands was lower than that in croplands and wetlands. Similarly, the average CUEe in global grasslands was lower than that in other ecosystem types (Chen et al., 2015b). In contrast, it was reported that the CUEe in grassland was higher than that in deciduous broad-leaved forestland and coniferous forestland, which contributes to the plant tissue in grassland yielding a lower investment in ecosystem respiration (Re) than that in forestland (Law et al., 2002). Our results demonstrated that the CUEe value in grassland was lower than that in forestland and cropland (**Figure 3**). This likely occurs because under the control of environmental conditions, grasslands are mainly distributed in semiarid and alpine areas, where the plant biomass is low and the active growth period is short. Compared to Re, the GPP was more significantly restricted by a low temperature and drought, which led to a higher Re/GPP ratio and thus a lower CUEe (1-Re/GPP) (Reichstein et al., 2007; Kato and Tang, 2008; Prescher et al., 2010; Chen et al., 2015b). Previous studies showed that there was a comparative Re in grassland to that in forestland and cropland, while the GPP was far lower than that in forestland and cropland (Chen et al., 2014; Chen et al., 2015a).

There were significant differences in the CUEe among the different EVAs. The CUEe on the Tibetan Plateau and agro-pastoral ecotones were significantly lower than those in the other EVAs (**Figure 3**), which may be related to the impacts of rainfall and temperature on productivity (Kato and Tang, 2008; Yu et al., 2013). In our study, the CUEe decreased with increasing rainfall, and it was also lower within the low-temperature range (**Figure 5**). Generally, rainfall on the Tibetan Plateau and agro-pastoral ecotones was higher than that in the other EVAs, but the temperature was lower (Liu et al., 2021), resulting in a low CUEe.

We found that the latitudinal distribution of the CUEe in EVAs in China was not significant (**Figure 4**), which was consistent with previous results (An et al., 2017). However, we found that the CUEe showed a decreasing trend with increasing longitude (**Figure 4**). In terms of longitude, the vegetation CUE usually decreased from west to east, which was closely related to ecosystem elevation (Chen and Yu, 2019). In EVAs in China, from west to east, the terrain transforms from plateau to plain terrains. Our results also showed that the CUEe increased with the elevation. This is consistent with previous studies on the vegetation CUE on a global scale (Zhang et al., 2009).

### Regulation mechanism of the CUEe in ecologically vulnerable areas in China

Climate factors such as the MAT and MAP are the two most important factors affecting the GPP, Re and NEP (Kato and Tang, 2008; Yu et al., 2013). Generally, with increaseing MAT and MAP, the GPP and Re increase, respectively, while the increase in the GPP is greater than that in Re (Yi et al., 2010; Tang et al., 2016). Therefore, the CUEe is expected to increase with increasing MAT and MAP. However, our results showed that the CUEe first decreased and then increased with increased MAT, and was negatively correlated with MAP (p<0.05) (**Figure 5**). The temperature is the most important factor affecting the carbon fluxes on the Tibetan Plateau (Kato et al., 2006; Saito et al., 2009), which is positively correlated with its GPP and Re. With increasing temperature, the rate of increase of Re was higher than that of the GPP; thus, the CUEe decreased instead. In plateau areas limited by water, a high temperature could inhibit the GPP and Re, and the rate of decrease of Re was higher than that of the GPP, so the CUEe increased with increasing temperature (Wang et al., 2014). Chen et al. (2019) found that the MAT could explain nearly 47% of the variation in the CUEe, and our results were similar (**Figure 5**). In contrast, the MAP explained the smaller differences in the CUEe (**Figure 6**). On a global scale, when the MAP is below 2300 mm, the CUEe shows a downward trend with increasing MAP (Zhang et al., 2009). However, in EVAs in China, rainfall is the main limiting factor (Hu et al., 2010; Dong et al., 2011). With increased rainfall, the GPP and Re increased, while the rate of increase of the Re was higher than that of the GPP, so the CUEe showed a decreasing trend with increasing rainfall.

Compared to the climate factors, the soil factors (soil pH and SOC) imposed a less notable impact on the CUEe (**Figure 6**). The variation range of soil conditions in EVAs in China may be limited (Zhang et al., 2019). Different ecosystems have different soil pH values, such as grasslands with high soil pH values and forestlands with low soil pH values (Chen and Yu, 2019). In our study, the CUEe was the lowest under almost neutral conditions (**Figure 5**). This suggests that an alkaline or acidic environment is not enough to yield the hightest CUEe value in EVAs. The variation range of the CUE of plants growing in poor-soil, low-temperature, drought-prone and other high-stress environments is generally larger than that of plants growing in suitable environments. Overall, among ecosystems, especially EVAs, the higher the SOC content is, the lower the CUEe (**Figure 5**).

The cycle of carbon between the Earth’s surface and the atmosphere is controlled by biotic and abiotic processes that regulate the storage of carbon in the biogeochemical cycle and release carbon into the atmosphere. The GPP and NEP are mainly determined by climate, soil and biotic factors. Therefore, it is not difficult for us to understand the regulatory mechanism of the CUEe. Through hierarchical partitioning analysis, we found that biotic factors such as the GPP, NEP and LAI exerted a greater impact on the CUEe in EVAs of China. However, the direct effects of climate (MAT and MAP) and soil factors (soil pH and SOC) were very limted in our study (**Figure 6**). We further explored the relationship among them through SEM (**Figure 7**). We propose that the geographical pattern shapes the climate and soil factors that influence vegetation factors such as ecosystem LAI and further determines the GPP and NEP, thus affecting the CUEe. Climate factors and soil factors mainly play an indirect role in determining the CUEe, while biotic factors play a more direct role in determining the CUEe (**Figure 7**). Based on our research, we found that the variation in the CUEe was mainly affected by climate, soil and biotic factors.

## Conclusion

This study integrated published literature on carbon fluxes data based on eddy covariance, and selected 55 flux sites among EVAs in China, including 3 forestland sites, 37 grassland sites, 6 cropland sites and 9 wetland sites. We preliminarily explored the spatial variation characteristics and influencing factors of the CUEe in EVAs in China. The study found that the average value of CUEe was 0.20, ranging from -0.39 to 0.67. There were significant differences in the CUEe among the different EVAs (p<0.05), but there were no significant differences in the CUEe among the different vegetation types (p>0.05). The CUEe showed a decreasing trend with increasing longitude, and its latitudinal distribution was not significant. We found that the CUEe first decreased and then increased with increasing MAT, soil pH and SOC, and decreased with increasing MAP. The most important factor affecting the CUEe were biotic factors, which directly affected the CUEe. However, climate and soil factors exerted indirect effects on the CUEe. In future research, plant physiological characteristics and soil nutrient availability features, such as soil carbon storage and nitrogen content, should also be considered to better understand the impact on the CUEe.

## Data availability statement

The original contributions presented in the study are included in the article/supplementary material. Further inquiries can be directed to the corresponding authors.

## Author contributions

ZL, GY, and ZC conceived of the article. ZL, MY, WZ, LH, TZ, ZC, and GY performed the statistical analyses. ZL, MY, WZ, LH, TZ, ZC, and GY drafted the manuscript. All authors contributed to the article and approved the submitted version.
